# Neuromuscular Consequences of an Extreme Mountain Ultra-Marathon

**DOI:** 10.1371/journal.pone.0017059

**Published:** 2011-02-22

**Authors:** Guillaume Y. Millet, Katja Tomazin, Samuel Verges, Christopher Vincent, Régis Bonnefoy, Renée-Claude Boisson, Laurent Gergelé, Léonard Féasson, Vincent Martin

**Affiliations:** 1 Université de Lyon, F-42023, Saint-Etienne, France and Exercise Physiology Laboratory, Jean Monnet University, Saint-Etienne, France; 2 HP2 Laboratory (INSERM), Joseph Fourier University and Exercise Research Unit, University Hospital, Grenoble, France; 3 Laboratory of Biochemistry, University Hospital Center Lyon-Sud, Hospices Civils of Lyon, Lyon, France; 4 Université de Lyon, F-42023, Saint-Etienne, France and Department of Anesthesiology and Intensive Care, University Hospital Center, Saint-Etienne, France; 5 Laboratory of Exercise Biology, Blaise Pascal University, Clermont-Ferrand, France; McMaster University, Canada

## Abstract

We investigated the physiological consequences of one of the most extreme exercises realized by humans in race conditions: a 166-km mountain ultra-marathon (MUM) with 9500 m of positive and negative elevation change. For this purpose, (i) the fatigue induced by the MUM and (ii) the recovery processes over two weeks were assessed. Evaluation of neuromuscular function (NMF) and blood markers of muscle damage and inflammation were performed before and immediately following (n = 22), and 2, 5, 9 and 16 days after the MUM (n = 11) in experienced ultra-marathon runners. Large maximal voluntary contraction decreases occurred after MUM (−35% [95% CI: −28 to −42%] and −39% [95% CI: −32 to −46%] for KE and PF, respectively), with alteration of maximal voluntary activation, mainly for KE (−19% [95% CI: −7 to −32%]). Significant modifications in markers of muscle damage and inflammation were observed after the MUM as suggested by the large changes in creatine kinase (from 144±94 to 13,633±12,626 UI L^−1^), myoglobin (from 32±22 to 1,432±1,209 µg L^−1^), and C-Reactive Protein (from <2.0 to 37.7±26.5 mg L^−1^). Moderate to large reductions in maximal compound muscle action potential amplitude, high-frequency doublet force, and low frequency fatigue (index of excitation-contraction coupling alteration) were also observed for both muscle groups. Sixteen days after MUM, NMF had returned to initial values, with most of the recovery process occurring within 9 days of the race. These findings suggest that the large alterations in NMF after an ultra-marathon race are multi-factorial, including failure of excitation-contraction coupling, which has never been described after prolonged running. It is also concluded that as early as two weeks after such an extreme running exercise, maximal force capacities have returned to baseline.

## Introduction

Walking and running are the most common modes of locomotion over the world in the daily life but these activities are also competitive events over a large range of distances. Running and walking for extreme durations, *i.e*. the so-called ultra-marathons, have become increasingly popular in the last few years throughout the world, particularly in the USA, Europe, Japan and South Africa. For instance, it was recently shown that the number of finishes in 161-km ultra-marathons increased exponentially in North America over the period 1977–2008 through a combination of an increase in the average annual number of races completed by each individual and an increase in the number of races organized every year [1]. Despite the recent success of ultra-endurance running, particularly in Europe, the physiological consequences of ultra-marathons are poorly understood. Investigating fatigue in ultra-marathons is a unique occasion to study human physiology as it is stretched towards it endurance limits [2], [3]. In recent years, only a few studies have been dedicated to this very unusual exercise. This is important because the aetiology of fatigue depends on the type of exercise [4], particularly intensity and duration. Other critical task variables include muscle groups involved, activation patterns and types of muscle contraction so the type of locomotion under consideration is also critical.

Neuromuscular fatigue is an exercise-related decrease in the maximal voluntary force or power of a muscle or muscle group, whether or not the task can be sustained. This potentially involves processes at all levels of the motor pathway from the brain to skeletal muscle. General discussions of potential factors involved in neuromuscular fatigue in the case of prolonged running exercises can be found elsewhere [2], [5], [6], [7]. Briefly, large central fatigue (*i.e.* reduced maximal voluntary activation) was observed after running 5 to 24 h [2], [6], [8], this activation deficit likely dependant on the muscle group tested, *i.e.* plantar flexors (PF) *vs.* knee extensors (KE) [2]. The implication of a central mechanism does not mean an absence of peripheral fatigue but seems to confine this type of alteration to a moderate level in the case of ultra-long exercise. Only one study has been conducted in exercise of extreme duration such as a 24 h-run [2]. This experiment involved level running (treadmill) so the combined effects of elevation (*i.e.* change in altitude over a course) plus extreme duration are unknown. Particularly, no study on prolonged running [7], [8], [9] - including the 24 h experiment [2] - has detected low frequency fatigue (LFF), which has been linked to E–C coupling alteration and muscle damage [10], [11]. Since evidence of muscle damage after prolonged running exists [12], this result was unexpected and led to the conclusion that a minimal exercise intensity is necessary to induce mechanical and metabolic disturbances that may promote the development of LFF [2]. Nevertheless, it can be hypothesized that the combination of extreme duration and large sections of downhill running (*i.e.* eccentric contractions of lower-limb extensor muscles) could be sufficient to induce LFF. Thus, the first purpose of the present study was to investigate the aetiology of KE/PF fatigue (neuromuscular function, blood parameters) in trained subjects performing a mountain ultra-marathon (MUM) characterized by an extreme distance/duration (166 km) and a large positive/negative elevation change (+/− 9500 m). Specifically, we hypothesized that neuromuscular function alterations, especially at the peripheral level, would be higher than after a running exercise of similar distance but on flat terrain [2]. To the best of our knowledge, no epidemiologic studies have been conducted on ultra-marathon runners, nor is experimental data available about the recovery processes following this type of extreme exercise. Specifically, it is currently unknown how much time is necessary to recover from such physical challenges. The second aim of the present study was to assess the recovery kinetics of neuromuscular function (NMF) by testing subjects 2, 5, 9 and 16 days after an international MUM.

## Methods

### Participants

Twenty-two male runners participated to this study after medical examination. Their main characteristics are given in Table 1. Thirty-four subjects were initially recruited but only 22 (*i.e.* 65%) were able to complete the MUM and took part to the first part of the study (fatigue). This finishers/starters ratio for the subjects of the present study was similar to the ratio when all race participants are considered (64%, data from the race organization). All the subjects were experienced ultra-marathon runners since to be registered to the MUM supporting the study (the North-Face Ultra-Trail du Mont-blanc 2009), one has to complete at least one MUM and one mountain marathon in the two years preceding the race. On average, the subjects had 13.3 years of training history in running and 5.0 years of ultra-endurance experience. Eleven of the 22 subjects participated to the second part of the study (recovery phase, see below). Their main characteristics, also presented in Table 1, are similar to the group of 22 subjects. There was no significant difference between the two groups for any of these characteristics.

**Table 1:** Main characteristics of the subjects.

	Age(y)	Height (m)	Mass(kg)	Body fat (%)	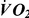 max(ml · kg^−1^ · min^−1^)	Training(h · wk^−1^)
Fatigue	40.2	1.78	73.4	12.6	54.1	9.0
(n = 22)	±7.4	±0.07	±6.4	±3.3	±4.3	±3.8
Recovery	37.9	1.78	76.0	13.3	55.0	7.7
(n = 11)	±7.2	±0.07	±6.5	±3.3	±4.8	±2.3

Data are mean values ± SD. Training was computed from May 1^st^ to the race day (end of August).

Twenty-two subjects performed only the pre- and post-race measurements (Fatigue) and 11 of these 22 participated to the whole experiment, *i.e.* PRE/POST/D+2/D+5/D+9/D+16 (Recovery).

### Ethics Statement

All subjects were fully informed of the procedure and the risks involved and gave their written consent. They were also allowed to withdraw from the study at will. The experiment was conducted according to the Declaration of Helsinki. Approval for the project was obtained from the local ethics committee (Comité de Protection des Personnes Sud-Est 1, France).

### Experimental design

**Figure 1:**
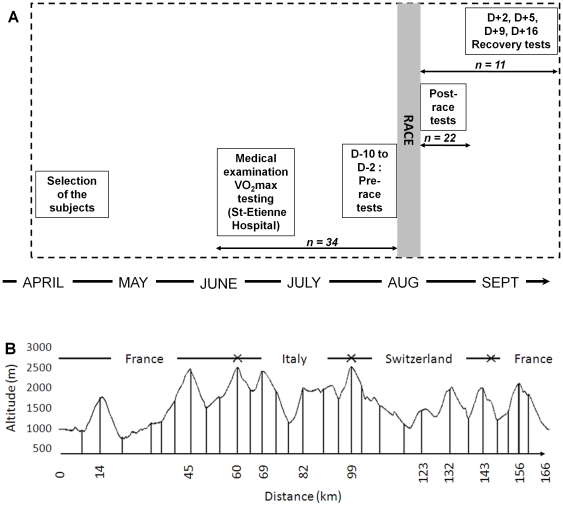
General view of the experiment and race profile. General view of the experimental testing (panel A) and the course of the race supporting the study (panel B).

An overview of the experiment is given in Figure 1A. The participants came into the laboratory three times for the subjects performing only the fatigue measurements (*i.e.* pre- and post-race, n = 22) and seven times for the subjects participating to the whole experiment (*i.e.* fatigue & recovery parts, n = 11). The first visit was performed 4 to 7 weeks before the second session (pre-race measurements) and consisted in a medical examination and determination of body mass, height and percentage of body fat (skinfold thickness measurements). During this first session, the subjects also performed a maximal test on a motorized treadmill (Gymrol S2500, HEF Tecmachine, Andrezieux-Boutheon, France), that aimed at determining anaerobic threshold, maximal oxygen uptake (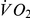
max) and the velocity associated with 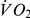
max (V
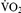
_max_). The initial velocity was set at 4 to 6 km · h^−1^ depending on the runner level of performance and the slope was +10%. The test consisted in a maximal continuous incremental test, the speed being progressively increased by 1 km · h^−1^ every 2 min. The last stage entirely completed was considered as V
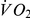
_max_. The highest 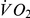
value (Douglas bag method) was considered as 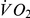
_max_. Detailed procedure of 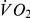
measurements can be found elsewhere [13]. During the first visit, the subjects were also fully informed regarding the experimental procedures. Particular attention was paid to familiarize them with the maximal voluntary contractions (MVC) and electrical stimulation of the KE and PF muscles. The subjects repeated trials of the procedures until they were able to produce consistent results.

The international race supporting the study was the North-Face Ultra-Trail du Mont-blanc 2009. About 2500 participants took part to the race in 2009. It consisted in running/walking 166 km with a total positive and negative elevation of 9500 m. The race characteristics (course profile, altitude) are detailed in Figure 1B.

**Figure 2:**
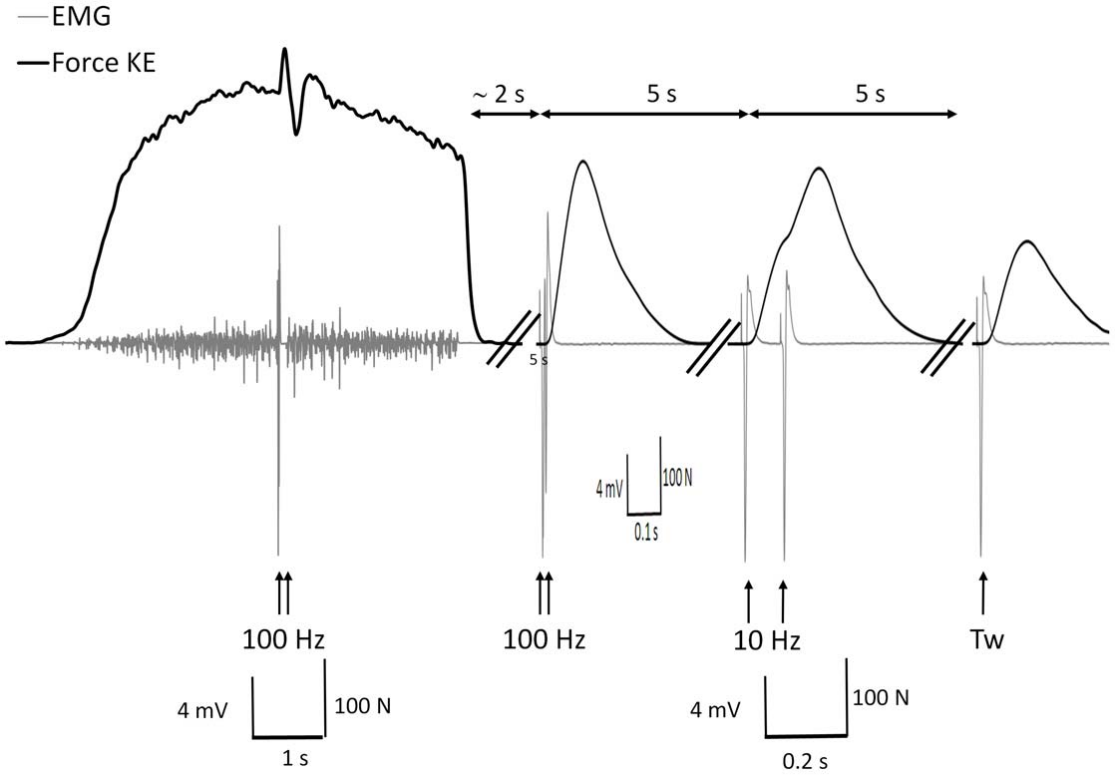
Typical torque and EMG traces during voluntary and electrically evoked contractions. Typical torque trace (black line) during the knee extensor maximal voluntary contraction and determination of maximal activation level, as well as high- and low frequency doublets (100 Hz and 10 Hz, respectively) and single twitch, after the mountain ultra-marathon. The black arrows indicate the timing of delivery of the stimuli. EMG is represented with a grey line.

The second (pre-race) an third (post-race) sessions, as well as sessions #4 to 7 performed 2, 5, 9 and 16 days after the race (D+2, D+5, D+9 and D+16, respectively) were nearly identical. The only difference was the fact that no warm-up was performed in session #3 (immediately after the race) for obvious reasons while 6-min cycling at about 50% 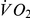
_max_ was done before NMF evaluation in the other sessions. The NMF was evaluated 2 to 10 days before the race (PRE), within 20±13 min after the race (POST) as well as at D+2, D+5, D+9 and D+16 for the 11 subjects who performed the second part of the study. After the delivery of 3 single electrical stimulations to evoke unpotentiated twitches, the NMF evaluation consisted in determining the isometric MVC of KE and PF to provide a global index of fatigue. Maximal voluntary activation levels of KE and PF was assessed using superimposed high-frequency (100 Hz) doublets to detect central fatigue. Finally, evoked stimulations were delivered to the relaxed muscle in a potentiated state to determine the extent and origin of peripheral fatigue (see below and typical trace in Figure 2). The set (MVC + superposed stimulation; high-frequency doublet, low-frequency doublet, single twitches) was repeated three times for both muscle groups. Incidental training (30 min to 1 h of light training per week) was performed by 7 of the 11 subjects between POST and D+9. Between D+9 and D+16, the subjects reported 74±84 min of light training. Body mass was measured without shoes immediately before race and at the beginning of the testing session at POST, D+2, D+5, D+9 and D+16.

#### Voluntary contractions

During all the MVCs, the subjects were strongly encouraged. For the KE testing, the subjects were seated in the frame of a Cybex II (Ronkonkoma, NY) and Velcro straps were strapped across the chest and hips to avoid lateral and frontal displacements. Subjects were also instructed to grip the seat during the voluntary contractions to further stabilize the pelvis. The KE muscles mechanical response was recorded with a strain gauge (SBB 200 Kg, Tempo Technologies, Taipei, Taiwan) located at the level of the external malleolus. Torque values were obtained from force measured by the strain gauge multiplied by the lever arm, *i.e.* knee-malleolus distance. All measurements were taken from the subject's right leg, with the knee and hip flexed at 90 degrees from full extension. PF muscles were tested with an instrumented pedal (CS1060 300 Nm, FGP Sensors, Les Clayes Sous Bois, France). For the PF testing, the subjects were seated in the frame of a Cybex II (Ronkonkoma, NY) similar to that used for KE. Velcro straps were also strapped across the chest and hips to avoid lateral and frontal displacements as well as across the forefoot to limit heel lift during the MVC. The hip, knee and ankle angles were set at 90 degrees from full extension.

#### Electrical stimulation

After femoral (for KE) and posterior tibial nerve (PF) detection with a ball probe cathode pressed into the femoral triangle and the popliteal fossa, respectively, electrical stimulation was applied percutaneously to the motor nerve via a self-adhesive electrode pressed manually (10-mm diameter, Ag-AgCl, Type 0601000402, Contrôle Graphique Medical, Brie-Comte-Robert, France). The anode, a 10×5 cm self-adhesive stimulation electrode (Medicompex SA, Ecublens, Switzerland), was located either in the gluteal fold (for KE) or on the patella (for PF). A constant current stimulator (Digitimer DS7A, Hertfordshire, United Kingdom) was used to deliver a square-wave stimulus of 1000 µs duration with maximal voltage of 400 V. The optimal stimulation intensity (45.0±9.7 mA; range: 30 mA to 65 mA on KE and 46.5±6.5 mA, 35 mA to 60 mA on PF) was determined from maximal twitch torque measurement (see below) and was reassessed before each session including POST. The stimulating intensity was supramaximal (150% of optimal intensity). For both KE and PF, stimulations included superimposed high-frequency doublets at 100 Hz (10-ms interstimulus interval) and a set of potentiated high-frequency doublet, low frequency doublet (10 Hz, 100-ms interstimulus interval) and single twitch (see Figure 2 for timing) delivered to the relaxed muscle in a potentiated state.

#### Electromyographic recordings

The EMG signals of the right vastus lateralis (VL) and soleus (SOL) were recorded using bipolar silver chloride surface electrodes of 10-mm diameter (Type 0601000402, Contrôle Graphique Medical, Brie-Comte-Robert, France) during the MVC and electrical stimulation. The recording electrodes were taped lengthwise on the skin over the muscle belly following SENIAM recommendations [14], with an interelectrode distance of 25 mm. The position of the electrodes was marked on the skin so that they could be fixed in the same place after the race and during measurements in the two-week recovery period. The reference electrode was attached to the patella (for VL EMG) or malleolus (for SOL EMG). Low impedance (Z<5 kΩ) at the skin-electrode surface was obtained by abrading the skin with thin sand paper and cleaning with alcohol. EMG data were recorded with PowerLab system (16/30 - ML880/P, ADInstruments, Bella Vista, Australia) with a sampling frequency of 2000 Hz. The EMG signal was amplified with octal bio-amplifier (Octal Bioamp, ML138, ADInstruments) with a bandwidth frequency ranging from 5 to 500 Hz (input impedance  =  200 MΩ, common mode rejection ratio  =  85 dB, gain  =  1000), transmitted to the PC and analyzed with LabChart 6 software (ADInstruments).

#### Blood samples

Peripheral venous blood samples were taken from an antecubital vein of participants before and immediately after completing the MUM as well as during recovery. Samples were drawn into nonadditive tubes under sterile conditions. Serum was separated from whole blood by centrifugation at 1,000 g for 10 min at room temperature. An OLYMPUS 2700 analyzer (Beckman Coulter, Brea, USA) was used for simultaneous assay with reagents from the manufacturer of sodium, potassium, total protein, urea, creatinine, calcium, glucose, C-Reactive Protein (CRP), Aspartate Aminotransferase, Alanine Aminotransferase, Creatine Kinase (CK) and Lactate Dehydrogenase (LDH). Myoglobin was measured by access immunoassay (Beckman Coulter, Brea, USA).

### Experimental variables and data analysis

#### M-wave

M-wave peak-to-peak amplitude and duration were considered. They were analyzed from the three single potentiated twitches evoked on the relaxed muscle.

#### Mechanical responses to nerve stimulation

The amplitude of the potentiated high frequency doublet (P_Db100_), the ratio of paired stimulation peak forces at 10 Hz over 100 Hz (Db10∶100) and the amplitude of the potentiated twitch peak torque (Pt) that followed the two doublets (see figure 2) were analyzed for both KE and PF. For these three parameters (P_Db100_, Db10∶100 and Pt), the average value computed from the three sets was considered. The use of Db10∶100 to assess LFF has been recently validated [15]. Potentiation was also calculated as the ratio of potentiated to unpotentiated Pt.

#### Maximal voluntary contractions and maximal activation level

The highest values of the three 5s-MVC was considered for each muscle group. During the voluntary contractions, electrical stimulations were superimposed to evaluate the level of activation. The technique was adapted from the twitch interpolation technique [16] and consisted in superimposing a high frequency doublet at supramaximal intensity on the isometric plateau. The control high-frequency doublet was delivered to the relaxed muscle 2 s after the end of the 5-s contraction. This provided the opportunity to obtain a potentiated mechanical response and so reduce the variability in activation level (%VA) values. The ratio of the amplitude of the superimposed twitch over the size of the control twitch was then calculated to obtain %VA as follows:
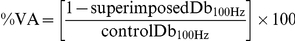



The RMS values of the VL and SOL EMG activity and average torque level were calculated during the best MVC trial over a 0.5-s period after the torque had reached a plateau and before the superimposed stimulation was evoked. This RMS value was then normalized to the maximal peak-to-peak amplitude of the M-wave (RMS⋅M^−1^).

#### Perceived exertion

The fatigue and pain sensations were measured PRE, POST and over the recovery sessions on a 10-cm visual analog scale. The subjects were requested to report their general fatigue, KE and PF pains and digestive system feeling.

### Statistics

All descriptive statistics presented in the text, tables and figures are mean values ± SD. Data were screened for normality of distribution and homogeneity of variances using a Shapiro-Wilk normality test and the Barlett's test, respectively. When conditions of analysis of variance (ANOVA) application were met, each study variable was compared between the different times of measurements using a paired t-test (PRE-POST, n = 22) or a 1-way (sessions: PRE-POST-D+2-D+5-D+9-D+16, n = 11) ANOVA with repeated measures for the subgroup who performed the recovery part of the study. Newhman-Keuls *post-hoc* tests were applied to determine between-means differences if the analysis of variance revealed a significant main effect. When conditions of application for parametric repeated-measures ANOVA were not met, Wilcoxon test (for fatigue) or Friedman ANOVA (recovery) was used. In that case, paired comparisons were done with Wilcoxon test with Bonferroni corrections. Pearson's product-moment correlation coefficients were also calculated between MVC decrease (average value of KE and PF) or peripheral fatigue and the following parameters: energy cost of running, performance, age and running experience. For all statistical analyses, a P value of 0.05 was accepted as the level of significance. The 95% confidence intervals (95% CI) were calculated for the relative changes from baseline in the main neuromuscular variables, *i.e.* MVC, %VA, P_Db100_ and Db10∶100. Effect size was also calculated for these variables using Cohen's d. The figure 0.2 was considered a small effect; 0.5, moderate; and 0.8, large.

## Results

### Performance, perceived exertion and maximal voluntary contraction

**Figure 3:**
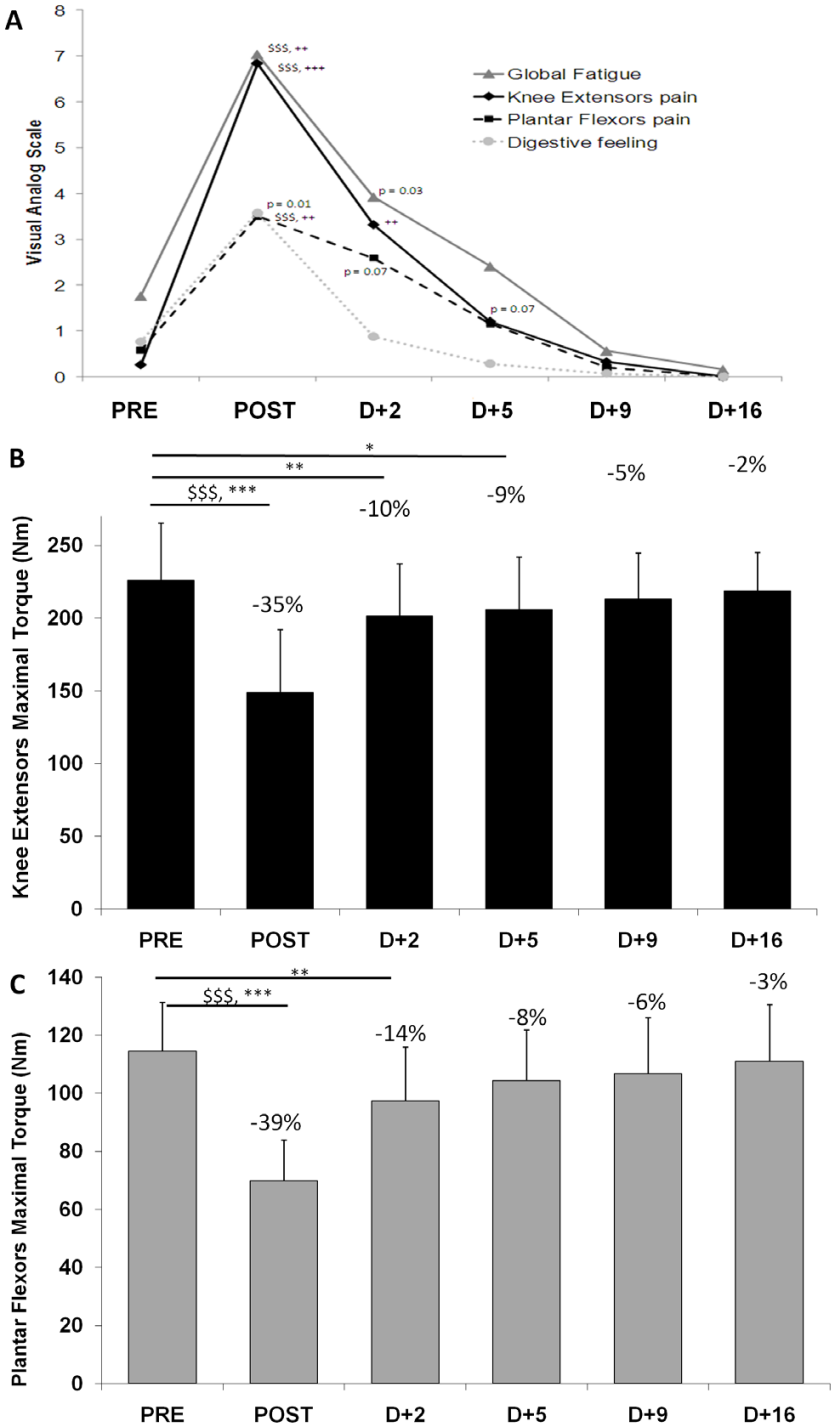
Self-reported general fatigue and maximal voluntary contraction on the knee extensor and plantar flexor muscles. Self-reported general fatigue, knee extensors (KE) and plantar flexors (PF) pain and digestive system sensations (panel A) as well as maximal voluntary contraction (MVC) on the knee extensor muscles (panel B) and on the plantar flexor muscles (panel C) before (PRE), after (POST) and 2, 5, 9 and 16 days after the race (D+2, D+5, D+9 and D+16, respectively). Data are mean values ± SD. $$$: P<0.001, Significance of t-test between PRE and POST (n = 22). ++: P<0.01; +++: P<0.001, Significance level of pairwise comparisons between PRE and every other measurements (n = 11) revealed by Wilcoxon test plus Bonferroni corrections when Friedman ANOVA was significant, P<0.01 to be significant. *: P<0.05; **: P<0.01; ***: P<0.001, Significance level of pairwise comparisons between PRE and every other measurements (n = 11) revealed by post-hoc analysis. Percentages indicated are for n = 11.

**Figure 4:**
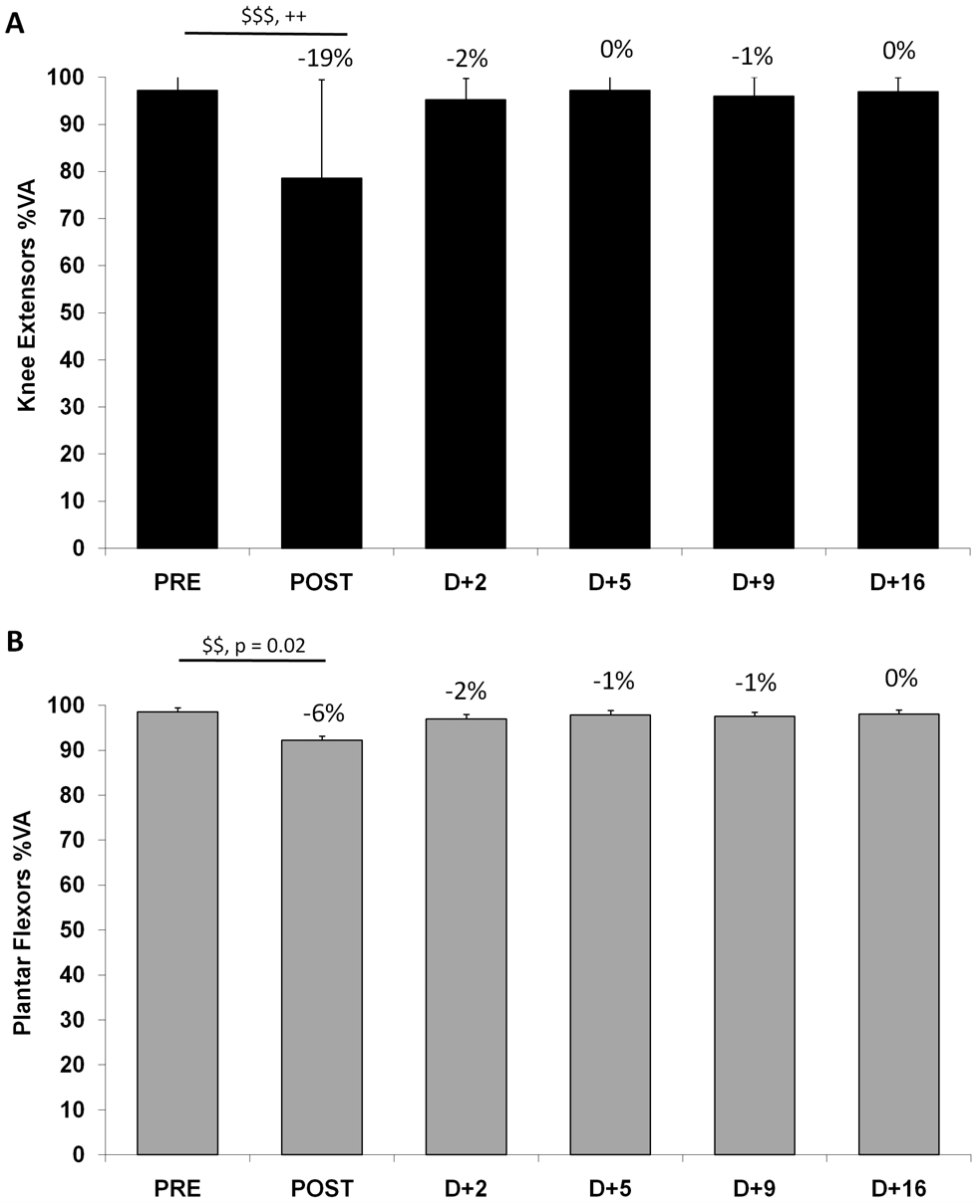
Maximal voluntary activation on the knee extensor and plantar flexor muscles. Maximal voluntary activation (%VA) of the knee extensor muscles (panel A) and the plantar flexor muscles (panel B) before (PRE), after (POST) and 2, 5, 9 and 16 days after the race (D+2, D+5, D+9 and D+16, respectively). Data are mean values ± SD. $$$: P<0.001, Significance of Wilcoxon test between PRE and POST (n = 22). ++: P<0.01: Significance level of pairwise comparisons between PRE and every other measurements (n = 11) revealed by Wilcoxon test plus Bonferroni corrections when Friedman ANOVA was significant, P<0.01 to be significant. Percentages indicated are for n = 11.

**Figure 5:**
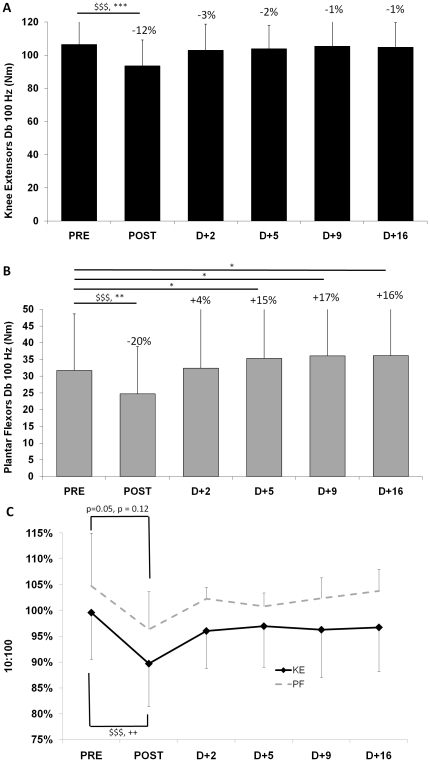
Mechanical responses to the high-frequency doublets and to the low-to-high frequency doublet ratio. Maximal high frequency doublet (Db 100 Hz) on the knee extensor muscles (panel A) and the plantar flexor muscles (panel B) as well as the low- to high-frequency doublet ratio (10∶100 ratio, panel C) before (PRE), after (POST) and 2, 5, 9 and 16 days after the race (D+2, D+5, D+9 and D+16, respectively). Data are mean values ± SD. Panels A and B: $$$: P<0.001, Significance of t-test between PRE and POST (n = 22). *: P<0.05; **: P<0.01; ***: P<0.001, Significance level of pairwise comparisons between PRE and every other measurements (n = 11) revealed by post-hoc analysis, are indicated by horizontal brackets). Panel C: $$$: P<0.001, Significance of Wilcoxon test between PRE and POST (n = 22). ++: P<0.01, Significance level of pairwise comparisons between PRE and every other measurements (n = 11) revealed by Wilcoxon test plus Bonferroni corrections when Friedman ANOVA was significant. Percentages indicated are for n = 11.

The average (± SD) finishing time of the subjects was 37 h 37 min±5 h 51 min. All levels of performance were represented in the group of subjects as shown by their rank ranging from 5^th^ to 1380^th^ place (of 1384 finishers). The subjects reported 15±25 min of sleeping time over the race. Poles were used by 95% of the subjects during uphills and 86% in dowhills. The self-reported general fatigue, KE and PF pain and digestive system sensations are given in Figure 3A. MVC declined significantly after the race for both muscles (P<0.001) by -35% ([95% CI: −28 to −42%], Cohen's d = 1.85) and −39% ([95% CI: −32 to −46%], Cohen's d = 2.88) for KE and PF, respectively. For all neuromuscular variables, the changes due to fatigue and recovery are presented in Figures 3, 4, and 5 and in Tables 2, 3, and 4 for the 11 subjects who have performed the recovery but statistics are presented for both the whole group (n = 22) and the subgroup (n = 11) that performed the recovery measurements. The average changes and effects size were similar when considered the whole group or the subgroup that performed recovery. For instance, for MVC changes for the 22 subjects were −32% ([95% CI: −27 to −37%], Cohen's d = 1.85) and −38% ([95% CI: −34 to −43%], Cohen's d = 2.88) for KE and PF, respectively. Most of MVC recovery occurred between POST and D+2, then MVC returned progressively to baseline values for both muscles (Figure 3B and 3C).

**Table 2:** Changes from baseline in potentiated peak twitch of knee extensors (KE) and plantar flexors (PF) and M-wave characteristics of Vastus Lateralis (VL) and Soleus (SOL) muscles.

	POST	D+2	D+5	D+9	D+16
Potentiated Peak Twitch					
KE	−22%$$$,***	−9%**	−5%^p = 0.07^	−5%^ p = 0.06^	−4%*
	[−28 to −17%]	[−13 to −4%]	[−10 to −1%]	[−9 to −2%]	[−10 to 1%]
PF	−20%$$$, ***	+4%	+15%*	+17%*	+16%**
	[−31 to −8%]	[−8 to 16%]	[−31 to 1%]	[4 to 29%]	[−27 to 5%]
M-wave Peak-to-peak Amplitude					
VL	−19%$$$,***	−19%***	−2%	+3%	+8%
	[−28 to −10%]	[−25 to −12%]	[−11 to 7%]	[−6 to 12%]	[−1 to 16%]
SOL	−20% $$, p = 0.09	−36%^ p = 0.02^	0%	+9%	−2%
	[−35 to −4%]	[−45 to −26%]	[−21 to 22%]	[−23 to 40%]	[−34 to 29%]
M-wave Peak-to-peak Duration					
VL	+14% $, p = 0.06	+10%^ p = 0.07^	+7%	+10%	+15%^ p = 0.05^
	[3 to 25%]	[3 to 17%]	[−1 to 16%]	[−2 to 21%]	[0 to 29%]
SOL	+12%^ p = 0.06^	+11%	+3%	−5%	0%
	[−4 to 29%]	[−6 to 27%]	[−13 to 20%]	[−18 to 7%]	[−12 to 12%]

Data are expressed as Δ changes from PRE values (n = 11). Data are mean values ± SD. 95% Confidence Intervals are given in square brackets.

POST/D+2/D+5/D+9/D+16 are the measurements performed immediately after and 2, 5, 9 and 16 days after the race.

$ : P<0.05,

$$ : P<0.01,

$$$ : P<0.001,

significance of t-test between PRE and POST (n = 22);

*: P<0.05;

**: P<0.01;

***: P<0.001,

significance level of pairwise comparisons between PRE and every other measurements (parametric tests).

+++ : P<0.001,

significance level of pairwise comparisons between PRE and every other measurement (nonparametric tests, P<0.01 to be significant). Δ changes from PRE values in potentiated twitch contraction time, potentiated twitch half-relaxation time and potentiation of knee extensors (KE) and plantar flexors (PF) are given in File S1.

**Table 3:** Main blood markers of muscle damage and inflammation.

PRE	POST	D+2	D+5	D+9	D+16
Creatine Kinase Activity (UI · L^−1^)					
124	15775$$$, ++	3276++	313++	108	116
±68	±17166	±3848	±263	±54	±49
Myoglobin (µg · L^−1^)					
28	1730$$$, ++	81++	41^p = 0.01^	24	25
±13	±1482	±63	±23	±11	±11
Lactate Dehydrogenase (UI · L^−1^)					
330	1448$$, ++	1002++	774++	469^p = 0.01^	369
±67	±1110	±594	±470	±228	±118
C-Reactive Protein (mg · L^−1^)					
2.0	46.8$$$, ++	30.0++	7.2++	2.5	2.3
±0.0^a^	±24.8	±19.7	±3.7	±1.2	±0.6
Leucocytes (mm^−3^)					
6218	10773$$$, ***	/	5440	/	/
±1359	±2473	/	±971	/	/
Creatinine (µmol · L^−1^)					
84.8	90.7$$	75.5**	78.2^p = 0.06^	79.5^p = 0.07^	79.8^ p = 0.12^
±13.0	±16.1	±13.2	±10.1	±14.0	±13.0

Data are mean values ± SD (n = 11). PRE/POST/D+2/D+5/D+9/D+16 are the measurements performed immediately before, after and 2, 5, 9 and 16 days after the race.

$$ : P<0.01,

$$$ :P<0.001,

significance of t-test between PRE and POST (n = 22).

**:P<0.01;

***: P<0.001,

significance level of pairwise comparisons between PRE and every other measurements (parametric tests).

++ : P<0.01,

+++ : P<0.001,

significance level of pairwise comparisons between PRE and every other measurement (nonparametric tests, P<0.01 to be significant).

a : For C-reactive protein, data <2 mg · L^−1^ were noted 2 mg · L^−1^. Values of total proteins, urea, aspartate aminotransferase and alanine aminotransferase as well as Δ changes from PRE values, Cohen's d and 95% Confidence Intervals are given in File S2.

### Central and peripheral components of fatigue and recovery

Maximal voluntary activation was significantly altered by the MUM for both muscles (Figure 4) by −19% ([95% CI: −7 to −32%], Cohen's d = 1.55) and −6% ([95% CI: 1 to −14%], Cohen's d = 0.92) for KE and PF, respectively. The development of central drive alterations was more pronounced in the KE muscle group than in PF and there was a significant correlation between %VA changes for KE and PF (R = 0.47; P<0.05). There was no more central fatigue at D+2 (Figure 4) for any of the two muscle groups tested. RMS ·M^−1^ values decreased with fatigue for KE (P<0.001) and were back to initial values at D+2 (data not shown). RMS ·M^−1^ did not change significantly for PF. Mechanical responses to evoked doublet at high frequency response are presented in Figure 5A and 5B for KE and PF, respectively. The MUM significantly altered P_Db100_ for both muscles by −12% ([95% CI: −7 to −17%], Cohen's d = 0.84) and −20% ([95% CI: −8 to −31%], Cohen's d = 1.19) for KE and PF, respectively. The recovery kinetics for P_ Db100_ were different among the two muscle groups. Indeed, while no significant difference between PRE and recovery values was observed for P_Db100_ from D+2 in KE, this response was increased (∼ +15%) from D+5 in PF. Figure 5C shows that low frequency fatigue (*i.e.* decrease of Db10∶100: −10% ([95% CI: −7 to −13%], Cohen's d = 1.14) and −8% ([95% CI: −3 to −12%], Cohen's d = 0.96) for KE and PF) was induced by the MUM and that Db10∶100 was back to initial values at D+2. Table 2 displays the main characteristics of the mechanical (KE and PF) and EMG (VL and SOL) responses to single electrical stimulations of the femoral and tibial motor nerves. In particular, this table shows clear differences between KE and PF in recovery kinetics for potentiated peak twitch and potentiation.

No significant correlations were found between global fatigue or peripheral alterations and age, level of performance or running experience.

### Blood analysis and body mass

**Figure 6:**
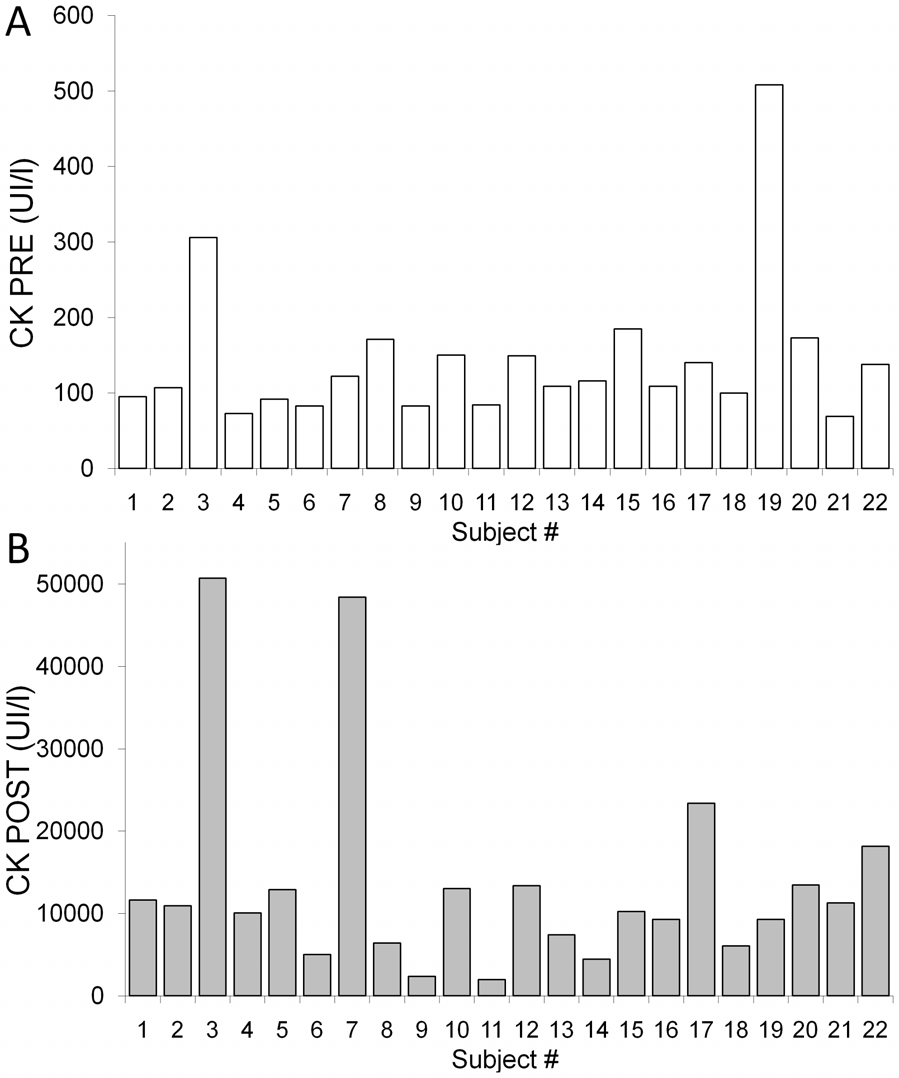
Individual creatine kinase activities. Individual creatine kinase activities (CK) before (PRE, panel A) and after (POST, panel B) the mountain ultra-marathon. Note the different scale.

The evolution of the main blood markers for muscle damage and inflammation are presented in Table 3. The broad range of individual CK values at POST is reported in Figure 6. Haematocrit concentration decreased from 44.3±2.5 at PRE to 39.8±3.0% at POST (P<0.001) and was back to initial values at D+5 (43.2±2.6%, not measured at D+2).

Changes in Na^+^, K^+^, Ca^2+^ and glucose concentrations are given in Table 4. Regarding body mass, the subjects lost ∼1 kg during the MUM (from 73.8±6.3 to 72.8 kg, P<0.001, n = 22 or from 75.1±6.2 to 74.3±5.9, P = 0.11, n = 11). The subjects' body mass went up at D+2 (75.6±6.3 kg, *NS* to compare with PRE), then returned to baseline values from D+5 (73.3±6.3, 74±6.3, 74.4±5.8 kg at D+5, D+9 and D+16).

**Table 4:** Sodium (Na^+^), potassium (K^+^), calcium (Ca^2+^) and blood glucose concentrations.

PRE	POST	D+2	D+5	D+9	D+16
Na^+^ (mmol · L^−1^)					
140.5	140.2	141.1	140.3	140.4	141.0
±1.4	±2.6	±2.6	±2.0	±1.9	±1.7
K^+^ (mmol · L^−1^)					
4.3	3.8$$$, ++	4.2	4.4	4.2	4.2
±0.3	±0.2	±0.3	±0.2	±0.2	±0.4
Ca^2+^ (mmol · L^−1^)					
2.51	2.40$$$, ***	2.30***	2.47	2.48	2.51
±0.04	±0.08	±0.07	±0.04	±0.07	±0.06
Glucose (mmol · L^−1^)					
4.9	7.6$$$, **	4.9	5.0	4.8	4.9
±0.5	±1.5	±0.9	±0.6	±0.6	±0.8

Data are mean values ± SD (n = 11). PRE/POST/D+2/D+5/D+9/D+16 are the measurements performed immediately before, after and 2, 5, 9 and 16 days after the race.

$$ : P<0.01,

$$$ :P<0.001,

significance of t-test between PRE and POST (n = 22).

**:P<0.01;

***: P<0.001, significance level of pairwise comparisons between PRE and every other measurements (parametric tests).

++ : P<0.01,

significance level of pairwise comparisons between PRE and every other measurement (nonparametric tests, P<0.01 to be significant).

a : For C-reactive protein, data <2 mg · L^−1^ were noted 2 mg · L^−1^. Δ changes from PRE values, Cohen's d and 95% Confidence Intervals are given in File S3.

## Discussion

The purpose of the present study was to investigate the aetiology of fatigue and the recovery kinetics of trained subjects performing exercise as extreme as a mountain ultra-marathon lasting ∼ 37.5 h. In particular, we hypothesized that neuromuscular function alterations, especially at the peripheral level, would be higher than after a running exercise of similar distance without elevation change [2]. The main results of the present study are: (i) the MVC decreases, which were by as much as 35–40% after the race, recovered within 9 days (−5–6%), (ii) the aetiology of fatigue was pluri-factorial and differed between knee extensors and plantar flexors, the main two muscle groups implicated in mountain ultra-marathons, (iii) low-frequency fatigue was detected for the first time after prolonged running exercise and (iv) most of the biological factors associated with tissue damage and inflammation had returned to normal 9 days after the race. Collectively, these results give the first picture of the physiological consequences of one of the most extreme exercises realized by humans in race conditions.

### Neuromuscular fatigue due to the mountain ultra-marathon

#### Central and peripheral alterations

The global NM fatigue for KE agrees with the strength loss–exercise duration relationship for running exercise [2], [5]: force loss dramatically increases as running duration increases for the first few hours, and then it tends to plateau for extreme durations. It is attractive to compare these results with our previous study investigating a 24 h treadmill run without slope [2] since the distance - but not the time due to elevation change of the MUM - was comparable (153 km *vs.* 166 km). Central fatigue has been consistently demonstrated as the main explanation for neuromuscular fatigue after ultra-endurance exercise on KE [2], [6] and a recent study suggested that this central drive reduction was not as high for PF as for KE after a 24 h treadmill run [2]. The present finding partially confirmed these observations since the decrease in %VA was the main cause of KE strength loss and PF was less affected by central fatigue (−19±20% *vs.* −6±12% for KE and PF, respectively, Fig 5A and 5B). Nevertheless, higher levels of central fatigue were expected in the present study due to the longer duration of exercise. Although the amplitude of central drive reduction was lower for PF muscles than for KE, there was a significant correlation between %VA changes for KE and PF, in line with the 24 h study [2]. This result could reflect the existence of a common central mechanism aimed at reducing neural input to working muscles to limit fatigue and damage [17], [18]. This safety mechanism may nevertheless be activated by peripheral feedback from muscle afferents [19] directly at the supraspinal level [20] or at the spinal level. Hyponatremia and hypoglycaemia were not observed among our subjects (Table 4). Thus, their implication in the generation of central fatigue can be excluded. Hyponatremia has been previously reported in prolonged running, probably because runners have been recently encouraged to overdrink during races [21]. Two factors could explain why this was not the case in the present race: (i) temperature was not as high as in other famous ultra-marathons like the Comrades or the Western States 100 miles thus runners probably did not drink as much and (ii) warm salted soups are served at every aid station and are usually appreciated by runners. Hypoglycaemia was not observed in the present study but glucose concentration changes must nevertheless be interpreted with caution since carbohydrate ingestion at the finish line was not controlled.

While intrinsic force (*i.e.* force induced by MVC + superimposed tetanus, [2]) was not assessed in the present study, large peripheral alterations have been evidenced by the alterations of the absolute high frequency doublet, M-wave alterations (lower amplitude and longer duration) and the existence of LFF. Peripheral alterations were more pronounced in the present study than in the 24 h run experiment, likely due to long downhill sections (*i.e.* eccentric contractions of KE and PF) during the MUM. LFF has been linked to failure of excitation-contraction coupling [11]. This result is interesting since we and others have not been able to detect any LFF after prolonged running, including ultra-marathons [2], [7], [8], [9]. This was unexpected since LFF generally occurs after exercise inducing muscle damage (*e.g.* [22]), which is the case after prolonged running [23]. As a result, we recently suggested that minimal exercise intensity is necessary to induce mechanical and metabolic disturbances that may promote the development of LFF [2]. The findings of the present study suggest that extremely long eccentric exercise (total negative elevation was higher than going from the top of Mount Everest to sea level in less than two days) at low intensity may be sufficient to trigger LFF, as is the case for much shorter distance but higher intensity (combination of negative slope and speed) downhill running [24]. Of note is that LFF existed in the present study and not in the 24 h run while plasma indexes of muscle damage (*e.g.* 13,633 *vs.* 13,319 UI · L^−1^ for CK) and global fatigue (strength losses) were comparable.

#### Knee extensor vs. plantar flexor fatigue

Contrary to what we expected from subjectively reported runners' experience, which was confirmed here by visual analog scale measurements (*i.e.* higher pain for KE than for PF, Figure 3A), strength losses were higher for PF than for KE, despite a relatively preserved activation (Figure 4B). Large peripheral fatigue was observed for PF, as demonstrated by the 20% decrease in P_db100_, a strong tendency toward LFF and M-wave alteration. It is interesting to note that while subjective pain was lower for PF, the slope of recovery for this variable was lower than for KE, suggesting long-lasting damage.

The relative contribution of PF and KE muscles probably also depends on the runner's level of performance and training background or the runner's technique. We hypothesized that, compared with a flat ultra-marathon [2], fatigue would be lower for PF because of elevation changes. In fact, it has been shown that the PF muscles are more active than KE during slow running on flat terrain, *e.g.* there was significantly greater muscle glycogen utilization in the calf than in the thigh [25]. The relative contribution of the two muscle groups in level *vs.* uphill running may however depend on the speed [26] so that the two vastii and the rectus femoris muscles are more active in uphill running at high speed, a situation not experienced by the runners during the MUM. Most of the uphills were actually performed walking during the MUM. For this type of locomotion, the major change due to slope is a greater contribution of hip extensors [27], a muscle group not studied in the present experiment. In addition, it is likely that the use of poles modifies the relative contribution of different muscle groups [28]. Since studies on marathon [29] and ultra-marathon [2] on flat surfaces reported higher MVC decrements for KE than PF, it was hypothesized [2] that KE was less resistant to fatigue than PF, potentially due to a lower percentage of type I fibers. The present data demonstrate that it is not a general rule.

#### Biological markers of fatigue

As expected, the MUM induced large effects on blood markers of muscle damage and inflammation (Table 3). Of particular interest is the large variability among subjects in these responses, *e.g.* in CK activity (Figure 6), which has already been observed [2].The comparison of these different markers with the literature shows that the MUM was extremely taxing. For instance, peak CK activities have been reported to be ∼4,500, ∼2,700 and ∼ 5000 UI · L^−1^ after races of 1600-km [30], 200-km [31] and 100 km [12], respectively. These are much lower than the values measured in the present study (∼13,600 UI · L^−1^ at POST; n = 22). Such activity levels are similar to those measured in patients undergoing severe rhabdomyolysis [32] but very rarely led to hospitalization. One exception was noted the year before the present experiment in the same race (North-Face Ultra-Trail du Mont-blanc®) when one subject went to an intensive care unit for dialysis [33]. CK activity was measured at 429 240 UI · L^−1^ in this subject but it was eventually found that extreme exercise was combined with the use of nonsteroidal anti-inflammatory drugs the week preceding the race and dehydration due to diarrhea. The two subjects with CK values above 45000 UI · L^−1^ (Figure 6B) did not have particular health troubles. The incidence of hospitalization may thus be secondary to inappropriate use of pharmaceutical. Nevertheless, we were able to get blood from one of our subjects who did not finish because he was stopped by a medical doctor of the race due to dark urine. His CK activity was 7126 UI · L^−1^ but his Mb concentration was 2308 µg · L^−1^. Interestingly, the markers of muscle damage and inflammation in the present study were lower than those consistently observed after the Spartathlon race, a 246-km continuous race from Athens to Sparta (Greece). In fact, while finishing times are similar for the Spartathlon and the North-Face Ultra-Trail du Mont-blanc®, higher concentrations of LDH, CK, CRP and total white blood cells have been reported after the Spartathlon compared with the present findings [32], [34], [35]. We hypothesized that the combination of surface (road, *i.e.* stiffer surface) and the fact that this race was run at a higher external temperature (up to 36°C) could explain these observations.

table-2-captionIn the present study, there was a significant decrease in [K^+^] and [Ca^2+^] after the MUM in the present study. This differs with the data of Martin et al. [2] where no significant change in plasma [K^+^] was observed after a 24 h run and with the data of Overgaard et al. [12] showing a significant increase in plasma K^+^ from 4.0 to 5.5 mmol · L^−1^ after a 100-km run. This discrepancy might be explained by the haemodilution shown by subjects having nearly 5% lower haematocrit concentration and the significant decrease of total protein at POST in the present study. This is in line with data showing that 4 days of prolonged exercise (5 h per day) induced a transient plasma volume expansion [36]. Large increases in plasma volume have also been reported after a 1600-km ultra-marathon [30], after a 24-h endurance race [37] and after an ultramarathon cycling [38]. However, such haemodilution was found neither after the Spartathlon [32] nor after the Comrades ultra-marathon, a 90-km road race in South Africa [39]. However, these races (i) were run at higher external temperatures, likely leading to dehydration [32] and (ii) were shorter, *i.e.* the runners were running faster and hence sweating more.

### Recovery kinetics

Ultra-marathons are increasingly popular but their physiological consequences and possible negative effects on runners' health are poorly understood. To the best of our knowledge, no epidemiologic data exist on ultra-marathon runners and experimental data about recovery after extreme duration exercise are extremely scarce. One major interest of the present study was thus to describe the recovery kinetics of neuromuscular function and blood markers over 16 days after the MUM. Most of the indexes of neuromuscular function evaluated in the present study recovered within 9 days after the MUM. Some exceptions are potentiated Pt and potentiation for KE, both factors still depreciated at D+16. There was also a tendency toward a longer vastus lateralis M-wave peak-to-peak duration at D+16. Even if recovery of neuromuscular function does not necessarily mean that functional capabilities have returned to normal since no information about endurance/metabolic properties are given in the present study, it is interesting to report that strength losses have been reported for much longer period after short- and intense eccentric exercise [*e.g.* [40], [41], mostly on upper body muscles] than after the MUM studied here.

Maximal voluntary activation was back to baseline values at D+2. This is in line with previous studies on different types of fatigue such as a prolonged (∼ 400 min) mountain marathon [42] or eccentric/stretch-shortening cycle exercise (e.g. [22]) where maximal activation was found to be back to baseline values as soon as one day post-exercise. It has been suggested that high central fatigue due to prolonged running is not due solely to central nervous system biochemical changes but that afferent fibers are probably involved [5] via direct or indirect inhibition from III-IV afferent fibers and disfacilitation of Ia afferent. It is known that group III-IV afferent fibers are sensitized by the production of pro-inflammatory mediators (*e.g.* [43]). It is thus tempting to speculate about the effects of the inflammation found in the present study (elevated serum CRP concentration and leucocytes concentration) on the activation of group III and IV afferent fibers and their potential effect on descending neural drive. However, our data are too limited to give a precise description of the inflammatory processes that might explain changes in central fatigue.

The recovery of peripheral fatigue showed different patterns for the two muscle groups and the different characteristics investigated here. M-wave was still depreciated at D+2 for both PF and KE. One hypothesis is that the EMG measurement was affected by oedema existing at D+2, as clinically observed and suggested by the significantly higher body mass at this time point.

Contrary to M-wave characteristics, LFF had recovered baseline values at D+2, suggesting that excitation-contraction coupling was not altered from a statistical point of view two days after the MUM. Forty-eight hours after a one-legged downhill run, severe eccentric exercise which induced marked LFF, Martin et al. [22] found that this variable had completely recovered. This result has since been confirmed for other eccentric exercise [44], [45] but no data exist for an exercise similar to the MUM. Raastad et al. [44] recently suggested that myofibrillar disruptions, rather than alteration-contraction coupling failure, seem to be a main cause for the long-lasting reduction in force-generating capacity after eccentric exercise. It is yet important to highlight here that potentiated twitch and potentiation were still depreciated at D+16 suggesting that the E-C was not completely back to normal 2 weeks after the MUM.

As tetanus was not evoked in the present study, it was not possible to assess the alteration and recovery of the intrinsic force-generating capacity of the muscle. However, it must be noted that the high-frequency doublets completely recovered at D+2 and stayed a pre-race values until D+16 (Figure 5A) for KE. An interesting result was that electrically evoked PF single twitch and doublets increased by about 15% from D+5 to D+16 (see Figure 5B and Table 2) while MVC was similar to PRE values for this muscle group at the same times. This was accompanied by a tendency toward a longer half-relaxation time for this muscle, suggesting a depreciated Ca^2+^ reuptake more than 2 weeks after the MUM. These surprising findings might also be linked to increased PF stiffness that could artificially increase mechanical responses to evoked single or double stimulations without necessarily affecting MVC. The fact that these increased evoked responses during recovery were limited to PF could be related to the higher peripheral fatigue for this muscle group compared with KE but muscle specificity in terms of tendon/muscle ratio cannot be ruled out. Altogether, this suggests that some intra-muscular mechanisms might not be completely normal. This surprising finding clearly requires further investigation.

The recovery kinetics of CK and LDH were in line with the literature [12]. It has been suggested that not only eccentric exercise but also prolonged running exercise [12] can lead to an increase in cytoplasmic Ca^2+^ and that elevated resting Ca^2+^ levels may activate proteolytic enzymes such as calpain to digest structural elements of fibers. In this context, it is important to report that plasmatic [Ca^2+^] was still reduced at D+2 even if this latter result may be linked to lower total proteins. In summary, no single neuromuscular step (from voluntary activation to contractile processes) can explain the lower MVC in the days after the MUM. It is likely that, as for neuromuscular fatigue, the recovery process is multi-factorial.

The purpose of this experiment was to document the physiological consequences of an exercise probably among the hardest performed all over the world in competition. It is concluded that the large alterations of neuromuscular function after an ultra-marathon race are multi-factorial and dependant on the muscle group in consideration. This study is the first to detect the existence of low-frequency fatigue after prolonged running, likely due to the 9500 m of negative change in altitude. As early as two weeks after such an extreme running event, maximal force capacities have returned to baseline but it is likely that neuromuscular measurements do not fully describe the recovery process of an athlete. We believe that the present paper is an important contribution but it must be seen as a first step toward comprehension of the physiological consequences of exercise as extreme as mountain ultra-marathons. Further works are now required to explain the influence of gender [46], age or weather conditions [47] in performance and odds of finishing in this type of extreme event. More experimental (focusing for instance on cartilage and tendon injuries) or epidemiologic studies are also required to understand whether the balance of risks/benefits is positive for this type of event and to help medical doctors and physiotherapists to assist athletes and coaches.

## Supporting Information

File S1 **Changes from baseline in potentiated twitch contraction time, potentiated twitch half-relaxation time and potentiation of knee extensors (KE) and plantar flexors (PF).**
(DOCX)

File S2 **Δ changes, Cohen's d and % Confidence Intervals for markers of muscle damage and inflammation.**
(DOCX)

File S3 **Δ changes, Cohen's d and % Confidence Intervals for Na^+^, K^+^, Ca^2+^ and blood glucose.**
(DOCX)
