# Supplementary material for: Neuromuscular Consequences of an Extreme Mountain Ultra-Marathon
Source: PLoS One. 2011 Feb 22;6(2):e17059. doi: 10.1371/journal.pone.0017059 (PMC3043077; doi:10.1371/journal.pone.0017059)
Supplement: File S1 — Changes from baseline in potentiated twitch contraction time, potentiated twitch half-relaxation time and potentiation of knee extensors (KE) and plantar flexors (PF). (DOCX) [file pone.0017059.s001.docx]

**Supplemental file 1.** Changes from baseline in potentiated twitch contraction time, potentiated twitch half-relaxation time and potentiation of knee extensors (KE) and plantar flexors (PF)

|  | | POST | D+2 | D+5 | D+9 | D+16 |
| --- | --- | --- | --- | --- | --- | --- |
| Potentiated Twitch Contraction Time | | | | | | |
| KE | +1% | | +5% | +4%% | +3% | +1% |
|  | [-5 to 5%] | | [1 to 10%] | [1 to 7%] | [1 to 5%] | [-2 to 5%] |
| PF | +1% | | +6% | +1% | +3% | +3% |
|  | [-7 to 9%] | | [2 to 10%] | [-4 to 7%] | [-2 to 8%] | [-1 to 8%] |
| Potentiated Twitch Half-Relaxation Time | | | | | | |
| KE | +4%^$^ | | +4% | +1% | -4% | 0% |
|  | [-2 to 10%] | | [-3 to 12%] | [-3 to 5%] | [-10 to 2%] | [-5 to 4%] |
| PF | +9%^$$^ | | +27%^***^ | +11% | +9% | +10% |
|  | [-1 to 20%] | | [15 to 39%] | [2 to 19%] | [-1 to 20%] | [0 to 20%] |
| Potentiation | | | | | | |
| KE | -14%^$$$,***^ | | -17%^***^ | -18%^***^ | -16%^***^ | -13%^***^ |
|  | [-20 to -8%] | | [-23 to -12%] | [-22 to -13%] | [-22 to -11%] | [-18 to -7%] |
| PF | -22%^$$$, ++^ | | -4% | +3% | +5% | +3% |
|  | [-28 to -16%] | | [-12 to 3%] | [-7 to 12%] | [-5 to 14%] | [-3 to 9%] |

Data are expressed as Δ changes from PRE values (n=11). Data are mean values ± SD. 95% Confidence Intervals are given in square brackets.

POST/D+2/D+5/D+9/D+16 are the measurements performed immediately after and 2, 5, 9 and 16 days after the race. ^$^: P < 0.05, ^$$^: P < 0.01, ^$$$^: P < 0.001, significance of t-test between PRE and POST (n = 22); ^*^: P < 0.05; ^**^: P < 0.01; ^***^: P < 0.001, significance level of pairwise comparisons between PRE and every other measurements (parametric tests). ^+++^: P < 0.001, significance level of pairwise comparisons between PRE and every other measurement (nonparametric tests, P < 0.01 to be significant).
