# Supplementary material for: Neuromuscular Consequences of an Extreme Mountain Ultra-Marathon
Source: PLoS One. 2011 Feb 22;6(2):e17059. doi: 10.1371/journal.pone.0017059 (PMC3043077; doi:10.1371/journal.pone.0017059)
Supplement: File S2 — Δ changes, Cohen's d and % Confidence Intervals for markers of muscle damage and inflammation. (DOCX) [file pone.0017059.s002.docx]

**Supplemental file 2.** Δ changes from PRE values, Cohen’s d and % Confidence Intervals for the main blood markers of muscle damage and inflammation.

|  | POST | D+2 | D+5 | D+9 | D+16 |
| --- | --- | --- | --- | --- | --- |
| **Creatine Kinase Activity** | | | | | |
| Cohen’d | 1,82 | 1,61 | 1,14 | 0,27 | 0,13 |
| Δ changes from PRE | 11744% | 2231% | 132% | -10% | -2% |
| CI | 16101% | 3233% | 194% | 7% | 6% |
|  | 7387% | 1229% | 70% | -27% | -10% |
| **Myoglobin** | | | | | |
| Cohen’d | 2,28 | 1,39 | 0,74 | 0,33 | 0,31 |
| Δ changes from PRE | 5728% | 193% | 44% | -14% | -11% |
| CI | 7605% | 295% | 62% | 4% | 3% |
|  | 3851% | 90% | 26% | -32% | -24% |
| **Lactate Dehydrogenase** | | | | | |
| Cohen’d | 1,90 | 2,03 | 1,65 | 0,94 | 0,42 |
| Δ changes from PRE | 326% | 194% | 128% | 26% | 10% |
| CI | 446% | 253% | 174% | 52% | 19% |
|  | 206% | 134% | 81% | 0% | 2% |
| **C-Reactive Protein** | | | | | |
| Cohen’d | 3,62 | 2,85 | 2,77 | 0,75 | 0,84 |
| Δ changes from PRE | 2241% | 1400% | 259% | 23% | 14% |
| CI | 2759% | 1811% | 337% | 48% | 27% |
|  | 1723% | 989% | 181% | -3% | 0% |
| **Total Proteins** (g · L^-1^) | | | | | |
| PRE | POST | D+2 | D+5 | D+9 | D+16 |
| 75.3 | 67.4^$$$, ***^ | 65.0^***^ | 72.5 ^p=0.10^ | 73.3 ^p=0.12^ | 74.3 |
| ± 3.3 | ± 3.7 | ± 2.6 | ± 4.0 | ± 3.2 | ± 4.6 |
| Cohen’d | 2,24 | 3,44 | 0,75 | 0,61 | 0,25 |
| Δ changes from PRE | -10% | -14% | -4% | -11% | -1% |
| CI | -9% | -12% | -2% | 1% | 0% |
|  | -12% | -15% | -5% | -24% | -3% |
| **Leucocytes** | | |  |  |  |
| Cohen’d | 2,37 | / | 0,67 | / | / |
| Δ changes from PRE | 109% |  | -14% |  |  |
| CI | 139% |  | 0% |  |  |
|  | 79% | / | -28% | / | / |
| **Urea** (mmol · L^-1^) | | | | | |
| PRE | POST | D+2 | D+5 | D+9 | D+16 |
| 6.3 | 11.1^$$$, +++^ | 6.2 | 6.1 | 6.6 | 6.3 |
| ± 1.5 | ± 3.0 | ± 1.9 | ± 1.5 | ± 1.4 | ± 1.4 |
| Cohen’d | 2,12 | 0,07 | 0,16 | 0,19 | 0,01 |
| Δ changes from PRE | 81% | 1% | -1% | -1% | 5% |
| CI | 105% | 15% | 10% | 14% | 20% |
|  | 57% | -13% | -12% | -16% | -9% |
| **Creatinine** | | | | | |
| Cohen’d | 0,41 | 0,71 | 0,58 | 0,39 | 0,38 |
| Δ changes from PRE | 7% | -11% | -7% | -15% | -5% |
| CI | 13% | -8% | -3% | -2% | 0% |
|  | 2% | -14% | -11% | -27% | -10% |
| **Aspartate Aminotransferase** (UI · L^-1^) | | | | | |
| PRE | POST | D+2 | D+5 | D+9 | D+16 |
| 26.7 | 551.4^$$$, ++^ | 235.7^++^ | 59.9^++^ | 32.2 | 26.0 |
| ± 4.1 | ± 536.5 | ± 202.1 | ± 32.5 | ± 9.7 | ± 5.8 |
| Cohen’d | 1,94 | 2,03 | 1,81 | 0,79 | 0,15 |
| Δ changes from PRE | 2018% | 792% | 122% | 11% | -3% |
| CI | 2928% | 1130% | 170% | 31% | 4% |
|  | 1108% | 454% | 73% | -8% | -10% |
| **Alanine Aminotransferase** (UI · L^-1^) | | | | | |
| PRE | POST | D+2 | D+5 | D+9 | D+16 |
| 17.2 | 67.6^$$$, ++^ | 73.4^++^ | 57.1^++^ | 36.2^++^ | 26.1^++^ |
| 4.0 | 56.0 | 48.0 | 31.0 | 15.9 | 11.5 |
| Cohen’d | 1,68 | 2,16 | 2,28 | 1,90 | 1,15 |
| Δ changes from PRE | 313% | 340% | 241% | 95% | 56% |
| CI | 470% | 471% | 328% | 146% | 90% |
|  | 156% | 208% | 153% | 43% | 22% |

POST/D+2/D+5/D+9/D+16 are the measurements performed immediately after and 2, 5, 9 and 16 days after the race.
