# Supplementary material for: Neuromuscular Consequences of an Extreme Mountain Ultra-Marathon
Source: PLoS One. 2011 Feb 22;6(2):e17059. doi: 10.1371/journal.pone.0017059 (PMC3043077; doi:10.1371/journal.pone.0017059)
Supplement: File S3 — Δ changes, Cohen's d and % Confidence Intervals for Na+, K+, Ca2+ and blood glucose. (DOCX) [file pone.0017059.s003.docx]

**Supplemental file 3.** Δ changes from PRE values, Cohen’s d and % Confidence Intervals for Sodium (Na^+^), potassium (K^+^), calcium (Ca^2+^) and blood glucose concentrations.

|  | POST | D+2 | D+5 | D+9 | D+16 |
| --- | --- | --- | --- | --- | --- |
| **Na^+^** | | | | | |
| Cohen’d | 0.18 | 0.27 | 0.16 | 0.09 | 0.29 |
| Δ changes from PRE | -0.2% | -0.3% | 0.4% | -0.2% | -0.1% |
| CI | -0.9% | -0.9% | -0.3% | -0.7% | -0.6% |
|  | 0.4% | 1.0% | 0.3% | 0.4% | 0.6% |
| **K^+^** | | | | | |
| Cohen’d | 2.02 | 0.50 | 0.07 | 0.43 | 0.45 |
| Δ changes from PRE | -12% | -3% | 1% | -12% | -3% |
| CI | -9% | 1% | 4% | 1% | 1% |
|  | -16% | -7% | -3% | -24% | -7% |
| **Ca^2+^** | | | | | |
| Cohen’d | 1.73 | 3.96 | 0.93 | 0.53 | 0.04 |
| Δ changes from PRE | -4% | -9% | -2% | -10% | 0% |
| CI | -2% | -7% | -1% | 2% | 1% |
|  | -6% | -10% | -2% | -23% | -1% |
| **Glucose** | | | | | |
| Cohen’d | 2.60 | 0.10 | 0.20 | 0.14 | 0.03 |
| Δ changes from PRE | 56% | 3% | 3% | -10% | 0% |
| CI | 70% | 12% | 8% | 4% | 7% |
|  | 42% | -7% | -2% | -24% | -7% |

POST/D+2/D+5/D+9/D+16 are the measurements performed immediately after and 2, 5, 9 and 16 days after the race.
